# Co-Design of an Intervention to Increase the Participation in Leisure Activities Including Adolescents with Cerebral Palsy with GMFCS Levels IV and V: A Study Protocol

**DOI:** 10.3390/jcm12010182

**Published:** 2022-12-26

**Authors:** Rocío Palomo-Carrión, Caline Cristine De Araújo Ferreira Jesus, Camila Araújo Santos Santana, Raquel Lindquist, Roselene Alencar, Helena Romay-Barrero, Elena Contell-Gonzalo, Karolinne Souza Monteiro, Elena Pinero-Pinto, Egmar Longo

**Affiliations:** 1 Faculty of Physiotherapy, University of Castilla-La Mancha, 45071 Toledo, Spain; 2 Hemi Child-Research Unit, University of Castilla-La Mancha, 45071 Toledo, Spain; 3 Rehabilitation Sciences Graduate Program, Faculty of Health Science of Trairi, Federal University of Rio Grande do Norte, Santa Cruz 59078-900, Brazil; 4 Department of Physical Therapy, Federal University of São Carlos, São Carlos 13565-905, Brazil; 5 Department of Physical Therapy, Faculty of Health Science, Federal University of Rio Grande do Norte, Campus Universitário Lagoa Nova, Natal 59076-740, Brazil; 6 Corseford School, Capability Scotland, Renfrewshire PA10 2NT, UK; 7 Department of Physical Therapy, Faculty of Nursery, Physiotherapy and Podiatry, University of Seville, 41004 Seville, Spain

**Keywords:** cerebral palsy, co-design, intervention, participation, involvement matrix, public and patient involvement, adolescents

## Abstract

The participation of adolescents with cerebral palsy (CP) within the community is reduced compared to their peers and is a barrier to their socialization, self-determination and quality of life. Patient and Public Involvement (PPI) is a key strategy for successful interventions, especially when involvement of the stakeholders takes place at all stages of the research. Co-design can be crucial for success as researchers, patients with CP and their families work together to bring the necessary elements to the interventions to be designed. The objectives will be: (1) To co-design an intervention aimed at improving the participation of adolescents with significant motor disabilities within the community in partnership with adolescents with CP, families and rehabilitation professionals. (2) To assess the feasibility of the co-design process in partnership with interested parties. The study will be based on Participatory Action Research (PAR) and will be held in Spain and Brazil. In both countries, the study will be carried out remotely with nine adolescents aged 12 to 17 years with CP, Gross Motor Function Classification System (GMFCS) levels IV–V, their families and six health professionals (physiotherapists and occupational therapists). Different dialogue groups will be created to involve adolescents, families and health professionals to the research’s project. To manage their involvement in the co-design process, the Involvement Matrix (IM) will be used, and according to the IM phases, four steps will be included in the research: (1) Preparation; (2) Co-design; (3) Analysis: results of the intervention protocol and the study’s feasibility and (4) Dissemination of results. Partnering with the public to design an intervention to improve participation can bring better results compared to protocols designed only by health professionals. In addition, it will allow for knowing the needs of adolescents with CP in terms of participation within the community. The study will also explore which roles were chosen by all participants and how they felt while actively participating in the process of co-designing an intervention protocol and their own perspectives on the use of the involvement matrix.

## 1. Introduction

Cerebral palsy (CP) is one of the most common physical disabilities, affecting approximately 2–3/1000 children [1]. It describes a group of permanent disorders that affect the development of movement and posture and contribute to activity limitations [1,2]. CP is a chronic health condition, which can increase the risk of developing problems related to mental health, chronic pain, fatigue and stress. These are highly comorbid in these patients, reducing their possibilities of interacting in the community [3]. Adolescents with CP experience limitations in their performance of day-to-day activities and restrictions on their participation in home, school and community life [4].

The International Classification of Functioning, Disability, and Health (ICF) by the World Health Organization (WHO) defines participation as a person’s involvement in life situations (WHO, 2001) [5]. Recently, participation has been understood from a Family of Participation-related Constructs (fPRC) perspective, which describes participation in terms of attendance (being there) and involvement (level of engagement) [6]. Participation is context-dependent and may predominantly be influenced by characteristics of the environment over characteristics of the individual, focuses on the societal level and is related to quality of life [7,8]. The participation of young people with CP within the community is reduced compared to their peers and is an impediment to socialization, self-determination and quality of life of the individual [8,9]. Participation depends on different factors such as children’s gross motor function and adaptive behavior for participation. Children’s ability to communicate and family support are important considerations for improving children’s social skills in life situations [10]. Thus, understanding the needs for participation within the community of adolescents with CP who have severe motor disabilities, giving them a voice through a collaborative process, can contribute to the success of the intervention.

A participative methodology facilitates democratic dialogue in the development and implementation of interventions and service improvement directed to that specific public, as they can share their real needs. Patient and public involvement (PPI) comprises the active involvement of patients and members of the public in the design and research process [11]. It aims to ensure that research is relevant to the intended audience and that their views are taken into account. PPI in research is currently being defined as “research being carried out ‘with’ or ‘by’ members of the public rather than ‘to’, ‘about’ or ‘for’ them” [12]. End-users are most often involved only in the early stages and/or in the final stages of research [13]. However, PPI can take place at any stage of the research process, from the development of the initial research questions through to specific aspects of study design, including data analysis and dissemination [11,12,13].

According to Bailey et al. (2015) [13], involving children and young people with disabilities in research is of vital importance as they are in an ideal position to opine on what works for them and their families. When dealing with young people with chronic diseases, researchers argue that the PPI improves the relevance and quality of projects and contributes to the personal development of this public. In addition, there seems to be a consensus that the involvement of young people with chronic diseases should become an integral and standard element of the projects that affect them. Van Schelven et al. (2020) [14] stated that these young people have always had a passive role in health and social assistance projects, as research subjects, recipients of an intervention and users of an instrument and that nowadays there is a growing consensus that they should be actively involved in matters that concern them.

In this sense, the Involvement Matrix (IM) [15] was developed to support conversation and discussion about roles and expectations, aiming for sustainable partnerships in research. This tool was jointly built by researchers and patients in the Netherlands to promote the collaboration of patients (from the age of 12) in projects and research. Using the IM ensures that the public is included in all phases of the research project and in an orderly manner, according to their preferences and interests [16]. The IM includes five roles for involvement (Listener, Co-thinker, Advisor, Partner, and Decision-maker) over three main phases of research projects (Preparation, Execution, and Implementation) [16,17]. This tool aims to support PPI in research projects, allowing different levels of involvement, which is useful to clarify the expectations during the process. It can be used prospectively to discuss strategies to be developed with the patients in different phases of projects, and retrospectively to discuss if the strategies were carried out satisfactorily [16,17].

Involving patients, caregivers and health professionals in the early stages of intervention development and evaluation is widely recognized as a good practice to elicit the opinions of users and professionals in order to create a credible and motivating program [17,18,19,20,21,22]. McDermott et al. (2010) [23] further state that the views of users and professionals are integral in the development of an intervention, which can help to clarify the mechanisms by which the intervention works, identify potential barriers to change, provide information about individual needs to users, and explore questions that can be used to develop and refine the intervention model.

Few studies [14,24] using a participative methodology have involved children and adolescents with more severe disabilities such as CP GMFCS level IV–V as active participants in research. In addition, it is known that interventions for children and adolescents with CP, especially at levels with greater dependence such as GMFCS levels IV and V, are strongly focused on components of the ICF such as function and structure, rather than activity, participation and contextual factors (physical environment, social environment, attitudinal environment, and personal factors unrelated to a child’s health condition). Thus, our objective will be to co-design an intervention to promote participation of young people with CP levels IV and V in partnership with the public and to assess the feasibility of the co-designing process in partnership with interested parties.

## 2. Materials and Methods

### 2.1. Study Design

The study will be based on the Participatory Action Research (PAR) [25], a term that encompasses numerous approaches to research in which researchers work collaboratively with stakeholders through an iterative cycle of fieldwork or practice, reflection, planning, research and action [12]. It is supported by a recommendation to execute research “with” people rather than “about” people. PAR is a qualitative research approach that seeks to maximize the participation of the people whose lives are researched about. It includes people affected by the research topic as researchers themselves [26]. To achieve this, young people with chronic conditions (cerebral palsy GMFCS levels IV–V), their families and health professionals will be involved. In this way, it is intended to be able to allow the public to whom the intervention is directed to decide on its execution based on their needs and expectations, obtaining results according to their perspectives and actually based on their own experiences [27]. Being able to involve the participants themselves in the initial stage of the research allows it to be targeted to their objectives and not those of the researcher. It gives validity and objectivity to the intervention by focusing on the user. In addition, involving families and health professionals allows for a greater awareness of the youth’s own reality. Thus, researchers and the public have a shared role and this allows the co-design of a viable intervention, whose objective is to make it possible to improve participation within the community [19].

### 2.2. Setting and Participants

This study was approved by the Ethics Committee of FIDMAG in Brazil (CAEE: 51319321.1.0000.5568) and Hermanas Hospitalarias (FIDMAG hospitable sisters Research Foundation) in Spain (PR-2022-07) according to the World Medical Association’s Declaration of Helsinki. Before the study began, the written informed consent of all participants: adolescents, families and pediatric physiotherapist and occupational therapist will be requested.

This research will be carried out remotely (online) in Brazil and Spain, with nine adolescents aged 12 to 17 years, diagnosed with CP, GMFCS level IV and V, their families and six health professionals (physiotherapists and occupational therapists) in each country. The sample size is based on research by Brooks et al. [28]. This study is also a co-design study involving youth with neuro-disability, parents and physicians. The sample they recruited has characteristics similar to those of this project. In this protocol, the sample will be recruited from different centers in Brazil and Spain.

#### Selection Criteria

##### Inclusion Criteria

Adolescents with CP from 12 to 17 years old, levels IV–V within the Gross Motor Function Classification System (GMFCS). They must have sufficient communication skills with support as needed, for example from using augmentative and alternative communication (AAC), help from another person or sign language translator. Other inclusion criteria are: participation of their family members, physiotherapists and occupational therapists who agree to participate in the study.

##### Exclusion Criteria

Adolescents who do not have the possibility of communicating with any AAC because there is no communicative interaction or functional communication, a problem answering questions and collaborating, other health conditions such as spina bifida or muscular dystrophy, and families, physiotherapists and occupational therapists who do not have time to participate in the meetings. Any participant who does not agree to sign the informed consent form will be excluded.

### 2.3. Recruitment

Recruitment will be intentional, as it is understood that the selection of participants in qualitative research often involves objective sampling, prioritizing the inclusion of information-rich cases from which much can be learned about issues of central importance. Importantly, purposeful sampling is highly adaptable and therefore applicable to many of the varied goals of engaging patients and the public as research partners.

Adolescents and their families will be recruited through known support networks, associations, philanthropic institutions and social networks. Rehabilitation professionals will be recruited for having previous experience working with the ICF participation constructs (the consultation of this information will be through publications, clinical background, ICF-attended courses or congress presentations by the professional).

### 2.4. Setting

The research will be carried out in Brazil and Spain, online, to facilitate the execution of the meetings. In this way, it will be possible to compare the participation of the same population in different countries, the influencing factors and how the approach works according to the perspectives of the parties involved in the research.

### 2.5. Assessment Tools

**Figure 1:**
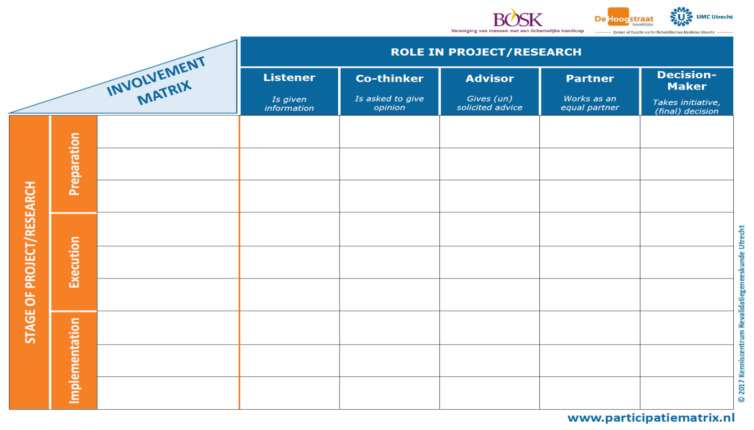
Involvement Matrix; Accessed on 1 March 2019, www.kcrutrecht.nl/involvement-matrix. © Center of Excellence for Rehabilitation Medicine Utrecht, used with permission.

To manage the involvement of the public in this research we will use the “Involvement Matrix” (IM) (Figure 1) with the adolescents, their families and the rehabilitation professionals [16].

**Figure 2:**
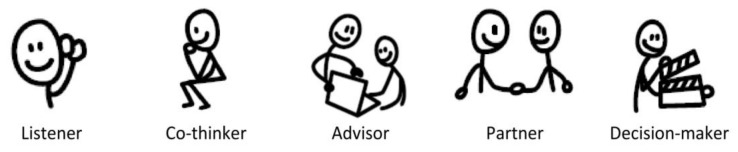
Illustration of the five roles for involvement in the Involvement Matrix. Accessed on 1 March 2019, www.kcrutrecht.nl/involvement-matrix. © Center of Excellence for Rehabilitation Medicine Utrecht, used with permission.

Initially, the tool will be translated into Spanish and Portuguese simultaneously and cross-culturally adapted for its proper use, as recommended by the Dutch team that developed the IM. This includes the five roles for involvement represented in Figure 2. When using the IM, all participants involved in the research project will fit into one of these roles, according to their preferences on each study phase [17]:

- Listener is a less active role but certainly not less important in the project;
- Co-thinker can also involve asking questions and giving feedback, as well as giving an opinion when asked;
- Advisor gives feedback from project leaders to patients on whether or not advice has been followed at any time;
- Partner is valuable not only at the start of a project but also at the intermediate and final phases. The partner has the same function as the main researcher;
- Decision-maker requires project leaders to have a ‘hands off’ attitude.

To evaluate the participation and the environmental barriers of adolescents in the community, we will use the Participation and Environment Measure for Children and Youth (PEM-CY) [29,30]. The PEM-CY is a parent-report instrument that examines participation and environment across three settings: home, school and community. It can be used to improve our understanding of the participation of children and young people with and without disabilities aged from 5 to 17 years and the environmental factors that support or hinder their participation in the home, school and community. It provides an overall environmental supportiveness score, as well as sub-scores that summarize the impact of particular features of the environment on participation in a given setting and the adequacy of available resources. For this research, only the community section of the PEM-CY will be used, considering the Spanish and Portuguese versions of the scale for the respective countries. A previously trained research assistant will pass the questionnaire to the adolescent and his/her caregiver so that the answers reflect the thinking of both, and not just the caregiver, as the scale recommends. The use of PEM-CY helps adolescents and their families to understand what it means to participate in the community, in addition to what can help or hinder participation.

#### 2.5.1. Qualitative Tools

Semi-structured interviews will be conducted with adolescents, families and health professionals, in order to identify their opinions on the intervention proposal, as well as on the design of the study aimed at the PPI in research. The interview guide for adolescents, families and health professionals is presented in Figure S1 (Supplementary Materials).

##### Research Phases

**Figure 3:**
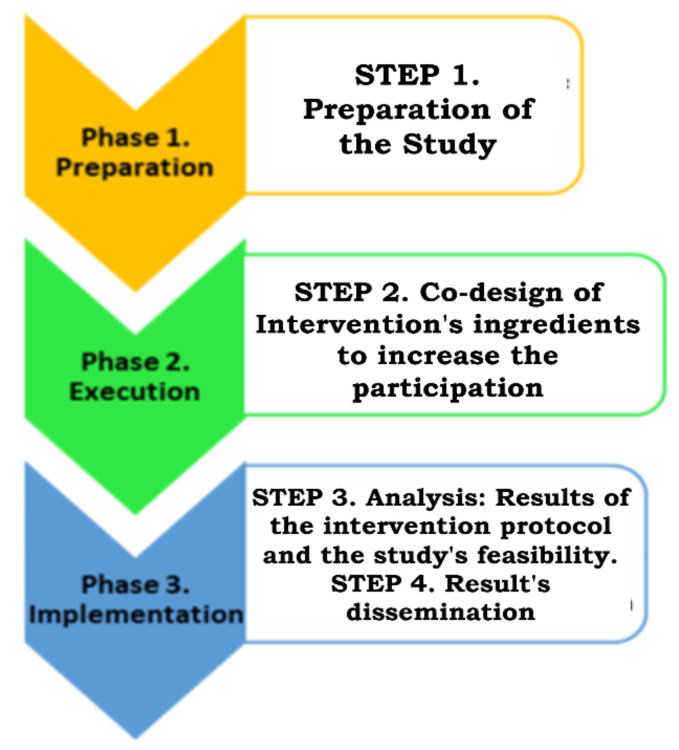
Research steps in the different phases of the Involvement Matrix.

This research will be conducted based on the phases suggested by the IM, having the following steps: preparation, co-design, analysis and dissemination of results (Figure 3) [17]. The objectives are related to the different phases included in the IM (phase 1 to phase 3). The main objective “To co-design an intervention aimed at improving the participation of adolescents with significant motor disabilities within the community in alliance with adolescents with CP, families and rehabilitation professionals” will be achieved from phase 1 to phase 2 of the IM, according to the Preparation and Execution parts in the IM.

To reach this objective we will answer the following research questions:

- What are the needs of adolescents with CP within the community?
- What barriers do you find in enhancing your participation in leisure activities?
- What are your perspectives on how to improve your participation?
- Do families and health professionals have the same perspectives on participation in the community as adolescents with CP?

The secondary objective “To assess the feasibility of the co-design process in partnership with interested parties” will be obtained in the last phase of IM, related to Implementation in IM. To reach this objective, we will answer the next research questions: Is it feasible to involve young people with CP, their families and professionals in the research? Is it possible to carry out their involvement in all phases of the study? Would the role in which they choose to become involved in the investigation be the right one?

#### 2.5.2. Phase (1) Preparation Phase-Step 1

##### (1.1) Involvement Matrix Translation

The preparation phase corresponds with Step 1 in the research project, PREPARATION. Phase 1 begins with the translation of the IM into Portuguese and Spanish and, later, its back-translation being later sent to the original authors to release the use of the tool in the research. The process will be similar and concomitant in both countries. The translations into Portuguese and Spanish will be performed by two pediatric physiotherapists independently. When all documents are ready, two versions will be analyzed by five pediatric physiotherapists and three online meetings will be performed to discuss the versions and consider changes to include in the final version to be understood in Spanish and Portuguese. When the meetings are over and we have the best and correct version for both countries, the final version will be back-translated into English by a pediatric physiotherapist. Finally, the back-translated version will be sent to the authors for checking and permission to use in both countries.

##### (1.2) Participants’ Recruitment

The recruitment of participants will be carried out intentionally, as explained in Section 2.1. Table 1 illustrates the details of this process in both countries and Table 2 shows the content and number of meetings that will be held with the different participants in the research project.

##### (1.3). Discussion/Development/Group Meetings (Shown in Figure 4)

A first meeting will be held for the presentation by the responsible researcher of the research project to all the participants, where we will use the *IM* in order to facilitate the discussion about what roles and responsibilities the participants would like to have within the project and how to achieve this in practice.

**Figure 4:**
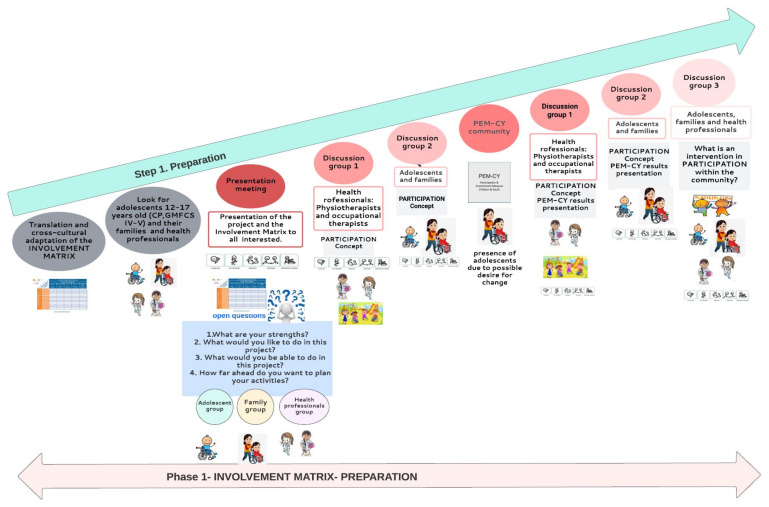
Step 1 of project’s research in phase 1 of the Involvement Matrix: PREPARATION.

Subsequently, focus groups will be organized to discuss the concept of participation focused on the ICF with the project participants, divided into three groups: adolescents, their families and health professionals. To support the participation of adolescents with CP in remote meetings, open questions will be established, asking them who wants to participate first so as not to generate rejection. If participation is not forthcoming, direct and more specific questions will be asked so that they can participate. In addition, if the adolescent wishes, the presence of a caregiver will always be allowed so that they can have the security and calm of being able of becoming involved in the meeting. They will be given enough time to communicate and their word will be respected.

A health professionals’ discussion group 1 will be created with all the health professionals to discuss the concept of PARTICIPATION. At first, they will be asked for their thoughts, and their understanding of the construct of participation (what they understand by “participation” according to their knowledge? What do they think about it? What do they know about it?). Afterwards, a dialogue will be opened between the attendees, using games and activities to create the understood concept of participation and building its definition. The Adolescents and Families (discussion group 2) will be created by adolescents and their families to reflect deeply and according to their own experiences on PARTICIPATION: What are their perspectives? How do they think it influences their condition? What barriers are present? As in the previous discussion group (health professionals), a dialogue will be performed to create the concept of participation and building its definition according to adolescents’ and families’ perspectives. In order to assess the participation and environment of the participating adolescents, the “community” session of the PEM-CY will be used [29,30]. This instrument considers the perception of parents and/or guardians to obtain information about involvement, frequency and desire for change in participation, as well as barriers and facilitators of the environment. To encourage the involvement of adolescents, researchers will be present online with families and adolescents when the questionnaire is answered by parents, to facilitate the inclusion of the adolescents’ voices if they have a desire for change.

After that, a meeting with health professionals (discussion group 1) will be held in order to present the result of the application of the PEM-CY questionnaire (community section). In addition, a meeting will be held to present these results with the adolescents and their families with the same purpose (discussion group 2). The presentation of the results of the PEM-CY (community section) will allow an understanding of what the restrictions of adolescents are in that context to analyze the barriers. Additionally, it could be possible to think of different strategies to favor their execution and how to design interventions to promote participation in the community.

At the end of the preparation phase, a meeting will be held with all participants (discussion group 3) in a focus group format, to think more easily about which ingredients should be part of the intervention protocol in terms of components, people involved and place of performance. It will provide a collaborative discussion about models of intervention to promote participation, taking components of the Pathways and Resources for Engagement and Participation (PREP) as an example [31]. PREP was developed in Canada and has been used as an evidence-based approach to enhance participation through modifying the environment, however, the research of Anaby et al. [32] includes adolescents with moderate motor impairment. Using PREP as an example will help adolescents, families and professionals to think more easily about which ingredients should be part of the intervention protocol in terms of components, people involved and place of performance [32,33].

#### 2.5.3. Phase (2) Execution Phase-Step 2 (Shown in Figure 5)

The Execution phase corresponds with Step 2 in the research project, CO-DESIGN. This phase is characterized by the stage where the participants will co-design an intervention protocol that encourages the participation of adolescents with CP GMFCS IV and V in leisure activities. It will initially consist of two brainstorming meetings (draft versions) where all participants will discuss and build necessary ingredients for an intervention protocol that will promote the participation of adolescents in the selected leisure activities. 

**Figure 5:**
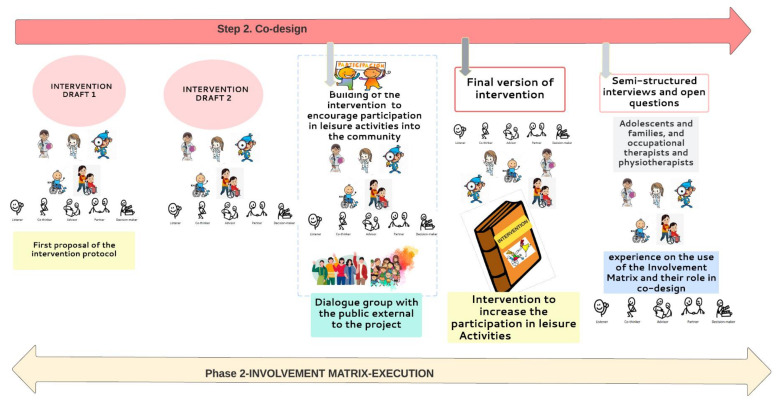
Step 2 of project’s research (co-design) in phase 2 of Involvement Matrix: EXECUTION.

##### (2.1) Intervention Checking

After the draft versions obtained in both meetings, a group external to the project (two adolescents, two family members and four health professionals, two OT and two PT) will be invited to express their opinion on the intervention protocol’s preliminary version.

After this stage, the intervention protocol proposal to increase the participation in leisure activities of adolescents with GMFCS IV and V will end with three meetings.

##### (2.2) Co-Design Evaluation

At the end of this phase 2, Execution in the IM, semi-structured interviews and open questions (Figure S1) for all participants will be carried out to identify the experience in the use of the IM and their roles in the co-design of the intervention protocol. To obtain information (co-design evaluation follow-up) from the intervention protocol application, to identify the strengths and limitations that occurred in its implementation, and to maintain their involvement in research and co-working to collect needs from the same population and other issues, different dialogue groups will be built to continue the discussion. These dialogue groups, constituted of adolescents with CP, families, health professionals and researchers will continue co-designing and co-working in relation to their own needs and to have a voice in the community, increasing the visibility of these young people and their families in society, as well as their engagement in research.

## 3. Analysis Procedure

### 3.1. Data Analysis of Co-Design Experiences and Role

The sample descriptive analysis will be performed through the SPSS statistical program [34]. For qualitative analysis, we will conduct an inductive thematic analysis of qualitative data collected in verbatim transcripts of audio recordings of meetings, materials used during the meetings and responses to open-ended questions to analyze members’ perceptions, barriers and facilitators to patient involvement in the co-design and in the IM use [35]. Analysis will be conducted during the course of the study by H.R.B using the N-Vivo software (QSR International, NVivo Qualitative Data Analysis Software) [36]. 

The content of the analysis will be shared with the participants of the process, so that they can give their opinion, suggest changes, and validate the results.

### 3.2. Participation of the Public in the Selection and Dissemination of Results and Analysis of the Feasibility of the Co-Design of the Study and Strategies of Dissemination

#### Phase (3) Implementation Phase-Step 3 and Step 4

##### Feasibility and Acceptability Analysis

Finally, the feasibility and acceptability (of PPI) on the participants’ involvement in the co-design of the final version of the intervention protocol will be analyzed, in the following aspects: evaluation of data collection (“How appropriate are the data collection procedures and purpose of the study?”). Follow-up questions address the participants’ ability to answer and be involved in the phases of the co-design (e.g., comprehension, capacity), the appropriateness of the amount of data collection, whether they are relatively complete and usable and if the use of the Involvement Matrix is appropriate to involve participants in a structured way and to co-design an intervention protocol; evaluation of acceptability and adequacy, of the study’s co-design and study’s procedures (“Are the study procedures suitable for and acceptable to participants?”). Meetings attendance, and engagement; time, capacity and understanding of the procedures and co-design; acceptability and satisfaction of the co-design to participants; evaluation of resources and the ability to manage and implement the study, adherence to the project (through attendance at meetings depending on the role acquired and execution of the phases and sub-phases corresponding to the IM), evaluation of the preliminary participants’ opinions on involvement in the study (“Does the research team have the resources and ability to manage the study and intervention?”). Follow-up questions address whether or not the research team has the space, administrative capacity, expertise, skills and time to conduct the study; ethics in implementing the study; budgetary considerations.

##### Dissemination Strategies (Shown in Figure 6)

The data analysis and dissemination will be carried out jointly with the research participants. To obtain information about the experience of involvement (strengths and limitations) in the intervention protocol, different dialogue groups will be formed. These dialogue groups, constituted of adolescents with CP, families, health professionals and researchers will continue co-designing and co-working in relation to their own needs and to have a voice in the community, increasing the visibility of these young people and their families in society, as well as their engagement in research.

**Figure 6:**
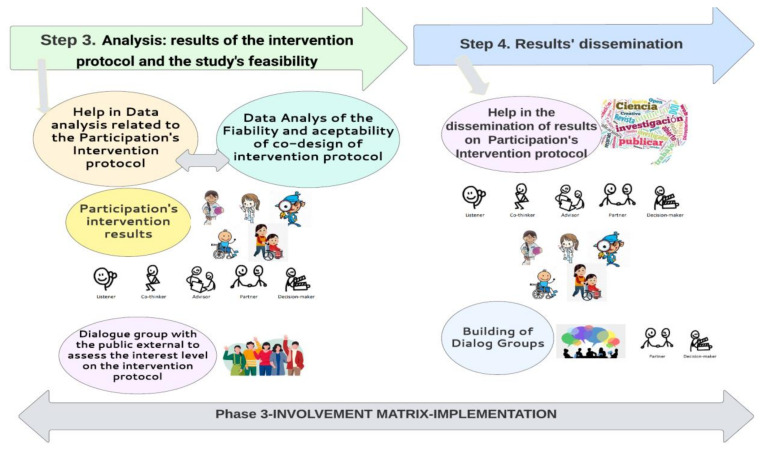
Steps 3 and 4 of project’s research in phase 3 of Involvement Matrix: IMPLEMENTATION.

The advisor, co-thinker and the researcher will select the most relevant results to be disseminated related to their interest and to give more support to adolescents who share the same condition (CP). The information will be analyzed and organized through the partners and decision-makers to be published in different ways: papers, websites, congresses. The results of semi-structured interviews, open questions and experience of the use of the involvement matrix and their role in the co-design will be disseminated according to their roles (partners and decision-makers). Adolescents with CP and their families will create dialogue groups to give support to the public with the same characteristics.

##### Coordination Brazil–Spain to Analyze the Data and Interpretation of Results from the Research Project

For the execution of both projects in Brazil and Spain, continuous coordination will be carried out between the responsible researchers on a weekly basis. For this, points of reflection will be established on which were the chosen roles, how the participants are involved in the meetings, structuring the meetings so that the same concepts can be addressed and can also be adjusted to the needs of the participants involved in each project. A comparison of the profiles that collaborate in the co-design is created in order to have information when interpreting the data and to see whether, therefore, personal characteristics can influence the planning, adherence and design of the protocol.

At the end of the project, the barriers encountered by the participants from Brazil and those from Spain will be compared. What were ingredients introduced to encourage community participation of adolescents for each country? Were there similarities in the choice? How was the implementation developed in each country.

## 4. Time Line

The study commenced in October 2022 with the first two tasks included in Figure 4, of Step 1 of Phase 1. An action schedule summarized in Table 3 is planned.

## 5. Discussion and Perspectives

The purpose of this paper is to present a protocol involving adolescents with CP, GMFCS levels IV–V, their families and health professionals. The aim is to co-design a preliminary protocol about the ingredients that should be included in the intervention to improve the participation of these adolescents in the community, considering various perspectives. Public and patient perspectives can be sought through their involvement and through participating in interviews or focus groups to provide data for others to analyze, interpret and act on [37].

PPI offers a methodology where researchers are in a continuous and reciprocal relationship with participants and make decisions with them about the research. The research is carried out with them and therefore satisfies their wishes, favoring its implementation in the population that shares the same condition [37,38]. This interaction can better ensure that research incorporates the participants’ voices including their priorities and preferences [39].

Researchers should involve the public and patients and to plan potential roles, responsibilities and tasks for their study as early as possible [39]. Therefore, the IM is a very useful tool that allows the participation of the public from an early stage in the design of the research project. In addition, it offers the possibility of choosing the role with which they want to get involved in the different stages of the design, creating learning opportunities for all participants according to their interest. It offers the opportunity to create an orderly and coordinated work together with the researcher and the participants to promote the co-design of the research [17,39].

The design of a preliminary intervention protocol that encourages the participation in leisure activities of adolescents with a chronic condition within the community will allow professionals and families from Brazil and Spain to consider their lived experiences. Using this study, it could be possible to guide the intervention based on their real needs, motivations and interests. The implementation in both countries will allow a comparison of the differences and similarities between both preliminary intervention protocols designed, the needs of some adolescents with CP regarding participation in the community, what were the roles chosen by all the participants, the relationships and dialogues established in the different dialogue groups created and the results obtained regarding the co-design of the intervention protocol and their own perspectives on the use of the IM [14,40,41].

The ultimate goal of an intervention should be to increase patient participation, but based on their own interests, hence the need to give them a voice. Considering that the study population has several factors present (communication problems, mobility, etc.) that limit their ability to be heard, it will allow them to feel motivated by entering the co-design of the research and will encourage their families and the health professionals to consider their needs. In addition, their active participation in disseminating the results will make it possible for the designed intervention protocol to be accepted by other young people with CP GMFCS IV and V and to create dialogue groups that give this group a “voice”, making it possible to improve life conditions. In order to increase the participation in most of adolescents with CP levels GMFCS IV–V, with profound intellectual disability (IQ < 25), or with communication impairments, we will facilitate their inclusion in research through different strategies through the results in this preliminary pilot study. The adolescents with severe disabilities will be able to watch images of the ingredients of the leisure activities chosen by the participants in this pilot study and videos of the adolescents performing the activities in the community, and we will record the behavior and reaction to these stimuli: facial and body response of motivation, rejection, etc., which will indicate their satisfaction or not with the activities. In addition, information will be collected with the help of their families in order to know their tastes regarding different leisure activities in the community and, therefore, to be able to co-design a program of ingredients of leisure activities that would increase their participation in the community.

As perspectives that will be obtained after this research project based on the PPI, the following topics could be included:

- What is the current participation of young people of adolescents with CP within the community like?
- Detection of barriers present in the community;
- Facilitators that increase participation: Improvements in access, adaptations and resources for participation according to the needs of young people with CP;
- Places within the community where the participation of young people with CP is encouraged (schools, leisure places, home, etc.);
- Role that the family and health professionals should have in the implementation of leisure activities within the community to encourage the participation of young people;
- Usefulness of the involvement of the PPI in the co-design of the intervention program to encourage the participation of young people with CP (GMFCS IV-V) within the community;
- Feasibility of co-designing the intervention protocol to encourage the participation of young people with CP (GMFCS IV-V) within the community;
- What should an intervention protocol to improve participation for adolescents GMFCS IV–V be like?

## Figures and Tables

**Table 1:** Recruitment process that will be addressed in both countries.

Country	Spain	Brazil
Places of recruitment	Fundació Aspace Catalunya em Barcelona (Aspace Foundation-Catalonia)	Associations, support networks and social networks
Type of meetings	Remote	Remote
Number of Adolescents with CP	9	9
Number of families of adolescents with CP	9	9
Health professionals: PTs	3	3
Health professionals: OTs	3	3

**Table 2:** Duration and content of the meetings that will be held in the research project in both countries.

Country	Spain	Brazil	Description	Duration of Each Group (min)
Presentation meeting	1	1	Presentation of the project and the IM to all stakeholders	60
Discussion groups-Participation	2	2	Presentation of the concept of participation based on the ICF and its meaning in practice	60–90
PEMCY questionnaire	9	9	Application of the PEM-CY community session	60–90
Discussion groups-Participation + PEMCY results	2	2	Discussion of the concept of participation now based on the results of the application of PEM-CY	60–90
Discussion Group-*What is a community participation intervention?*	1	1	Discussion about what is an intervention aimed to improve the participation of young people with disabilities in the community	60–90
Discussion Group-intervention draft	2	2	Co-design of an intervention draft aimed at the participation of young people with CP GMFCS IV-V in leisure activities	60–90
Meetings with the external public	1	1	Presentation of protocol results so far and capture criticism and suggestions from an external group made up of adolescents with CP GMFCS IV and V	60
Discussion Group-final version	3	3	Co-design of final version with all ingredients that should be present in the intervention for CP to increase the community participation	60–90
Semi-structured interviews and open-ended questions	24	24	A guide with questions will be used	60–90

**Table 3:** Anticipated time line of the project.

		Oct–Dec 2022	Jan–Mar 2023	Apr–Jun 2023	Jul–Sep 2023	Oct–Dec 2023	Jan–Mar 2024
Phase 1. Step 1		X	X				
Phase 2. Step 2				X	X		
Phase 3	Step 3					X	
	Step 4						X

X: Execution time of the phase/step corresponding to the research project.

## Supplementary Materials

The following supporting information can be downloaded at: https://www.mdpi.com/article/10.3390/jcm12010182/s1, Figure S1: Open questions for participants.
