# Supplementary material for: Co-Design of an Intervention to Increase the Participation in Leisure Activities Including Adolescents with Cerebral Palsy with GMFCS Levels IV and V: A Study Protocol
Source: J Clin Med. 2022 Dec 26;12(1):182. doi: 10.3390/jcm12010182 (PMC9821276; doi:10.3390/jcm12010182)
Supplement: Supplementary file 1 [file jcm-12-00182-s001.zip › jcm-2062042-supplementary.pdf]

Supplementary Material. Open questions directed to adolescents, families and health professionals related to their involvement in the co-design and their roles.

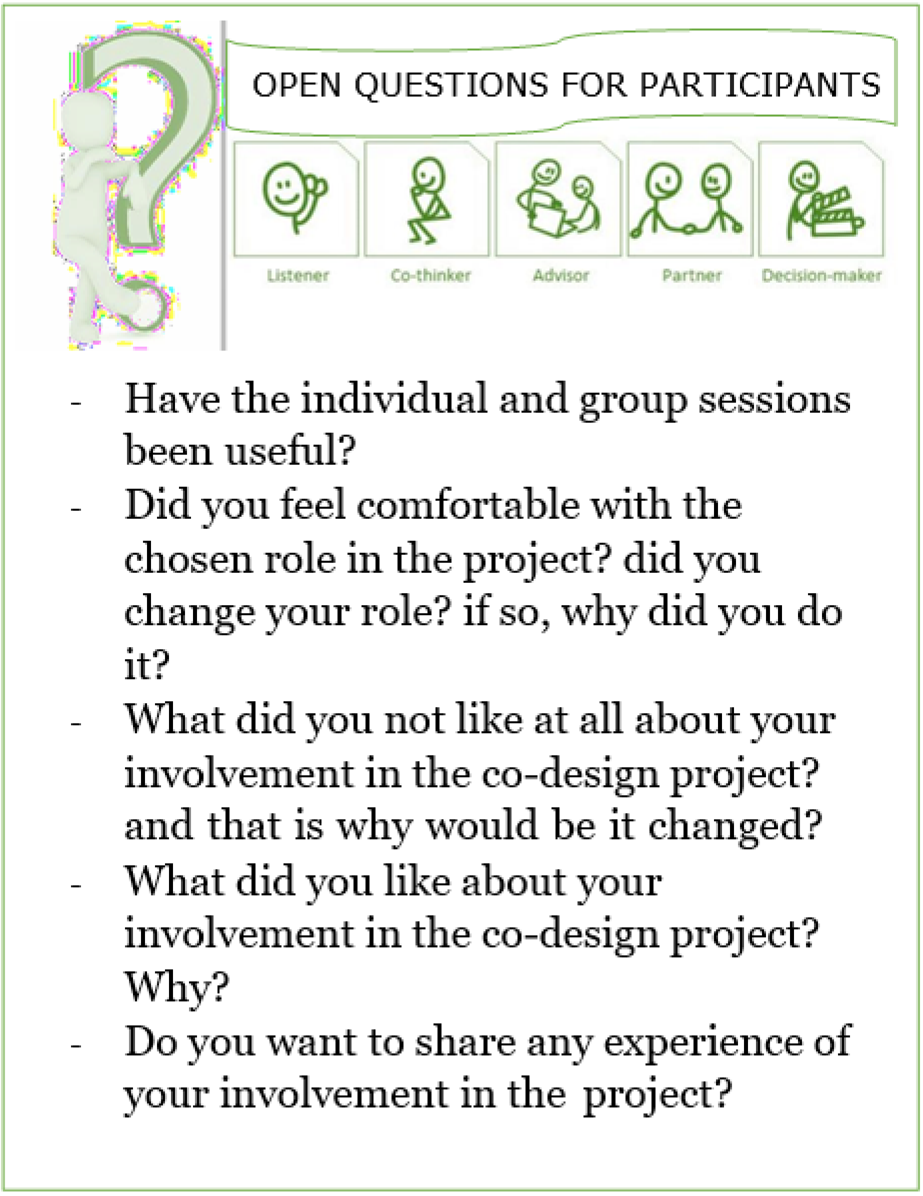

Figure S1. Open questions for participants.
